# *TNF-α Gene* Knockout in Triple Negative Breast Cancer Cell Line Induces Apoptosis

**DOI:** 10.3390/ijms14010411

**Published:** 2012-12-24

**Authors:** Valentina Pileczki, Cornelia Braicu, Claudia D. Gherman, Ioana Berindan-Neagoe

**Affiliations:** 1 Faculty of Pharmacy, “Iuliu Hatieganu” University of Medicine and Pharmacy, 4 Pasteur Street, Cluj-Napoca 400349, Romania; E-Mail: valentinapilecki@gmail.com; 2 Department of Functional Genomics and Experimental Pathology, Cancer Institute “Ioan Chiricută”, 34–36 Republici Street, Cluj-Napoca 400015, Romania; E-Mails: cornelia.braicu@iocn.ro (C.B.); ioanaberi@iocn.ro (I.B.-N.); 3 Research Center for Functional Genomics, Biomedicine and Translational Medicine, “Iuliu Hatieganu” University of Medicine and Pharmacy, 8 Victor Babes Street, Cluj-Napoca 400012, Romania; 4 Surgical Clinic II, 4–6 Clinicilor Street, Cluj-Napoca 400006, Romania; 5 Department of Surgery, “Iuliu Haţieganu” University of Medicine and Pharmacy, 8 Victor Babes Street, Cluj-Napoca 400012, Romania

**Keywords:** RNA interference, apoptosis, cell signaling pathways, gene therapy

## Abstract

Tumor necrosis factor alpha (TNF-α) is a pro-inflammatory cytokine involved in the promotion and progression of cancer, including triple negative breast cancer cells. Thus, there is significant interest in understanding the molecular signaling pathways that connect TNF-α with the survival of tumor cells. In our experiments, we used as an *in vitro* model for triple negative breast cancer the cell line Hs578T. The purpose of this study is to determine the gene expression profiling of apoptotic signaling networks after blocking TNF-α formation by using specially designed siRNA molecules to target TNF-α messenger RNA. Knockdown of *TNF-α* gene was associated with cell proliferation inhibition and apoptosis, as observed by monitoring the cell index using the xCELLigence RTCA System and flow cytometry. PCR array technology was used to examine the transcript levels of 84 genes involved in apoptosis. 15 genes were found to be relevant after comparing the treated group with the untreated one of which 3 were down-regulated and 12 up-regulated. The down-regulated genes are all involved in cell survival, whereas the up-regulated ones are involved in and interact with pro-apoptotic pathways. The results described here indicate that the direct target of TNF-α in the Hs578T breast cancer cell line increases the level of certain pro-apoptotic factors that modulate different cellular networks that direct the cells towards death.

## 1. Introduction

Triple negative breast cancer is a highly aggressive subtype that is frequently observed in young patients (<35 years) with a poor overall survival rate [1]. The name *triple negative* comes from the lack of cell membrane receptors for estrogen, progesterone and HER2. Its biology signature reveals critical alterations of molecular pathways implicated in cell cycle, DNA repair, NF-κB signaling, inflammatory response and angiogenesis [2,3].

Tumor necrosis factor-α (TNF-α) is a pro-inflammatory cytokine involved in the promotion and progression of cancer [4]. It plays an important role in the tumor microenvironment both as a membrane-integrated protein and in its soluble form generated after proteolytic cleavage [5]. TNF-α stimulates many signaling pathways by binding to two receptors, TNFR1 (p55) and TNFR2 (p75) [5,6]. TNF receptor activation leads to the activation of multiple cell signaling cascades that lead to inflammation and survival of the tumor cells.

Several studies have suggested that TNF-α plays an important role in the molecular events that link inflammation with development and evolution towards breast cancer [7]. At the same time, more and more studies connect TNF-α expression in triple negative breast cancer with the blockage of estrogen and progesterone receptors, increasing the poor prognosis of the patients with this subtype of breast cancer [8]. Thus, there is significant interest in understanding the molecular signaling pathways that connect TNF-α with the aggressive behavior of triple negative breast cancer. In our study, we intend to determine the gene expression profiling of signaling networks after blocking NTF-α formation by using special designed siRNA molecules to target TNF-α messenger RNA.

The small interfering RNA class or siRNA became an important tool for functional genomics studies and has great potential in obtaining new and efficient instruments for treating human disease [9]. Synthetic small interfering RNA oligomers efficiently exploit the cells’ natural occurring mechanism of RNA interference to conduct gene regulation. siRNAs are exogenous, small double-stranded RNAs of 19–28 base pairs [10] that are broadly used in molecular biology for their control of gene expression [11,12] through the activation of a specific protein complex known as the RNA-induced silencing complex (RISC) [13]. This will result in the specific knockdown of a target gene. By the incorporation of the siRNA molecule that is complementary to the corresponding sequence of a specific messenger RNA, the RNA interference machinery disrupts the formation of a specific protein [14]. Therefore, by controlling the translation of disease-associated genes, RNAi becomes an important and powerful approach for developing new therapeutics against a wide range of human diseases [15].

siRNA has broad applications in the field of functional genomics, helping scientists to study the implication of certain genes in cell signaling pathways. Based on the great results obtained in certain laboratories on culture cells and animal models [16], the next step is to develop new means for cancer therapy [17]. siRNA can provide an oasis of hope for the patients bearing tumors that do not respond to conventional treatment, like triple negative breast cancer.

## 2. Results and Discussion

### 2.1. Cell Survival, Proliferation and Migration after Treatment with siRNA-TNF-α

**Figure 1:**
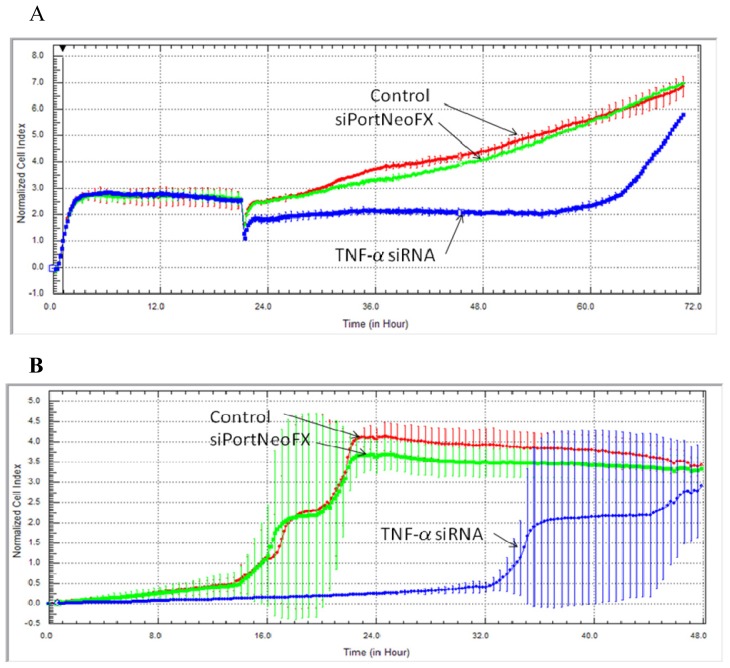
Time kinetics of cell growth (**A**) and migration (**B**) after *TNF-α* knock-down with the RTCA System.

The growth rate was measured by the xCELLigence RTCA (real-time cell analysis) System. These non-invasive cell-based assays provide physiologically relevant data in the case of treatment with siRNA-TNF. Knockdown of TNF-α gene was associated with a cell proliferation inhibition, as we can see from the electronic readout of cell sensor impedance that is translated as the reduction of the cell index (Figure 1) when compared to untreated cells (control) or to the transfection agent (siPort NeoFX).

After 72 h of monitoring cell proliferation with the xCELLigence System, we can observe a continuous decrease in cell growth and motility after treatment with siRNA-TNF. In the last years, TNF-α was associated with the stimulation and expression of the *NF-κB* gene, an important modulator that activates many genes implicated in cell survival, growth and expression of pro-inflammatory cytokines, including TNF-α, which acts as a positive feedback signal to stimulate and continuously support cell growth [14]. This is in correlation with the well known fact that tumors secret TNF-α [14,16].

### 2.2. Apoptosis/Necrosis in the Hs578T Cell Line after Blocking the Expression of *TNF-α*

**Figure 2:**
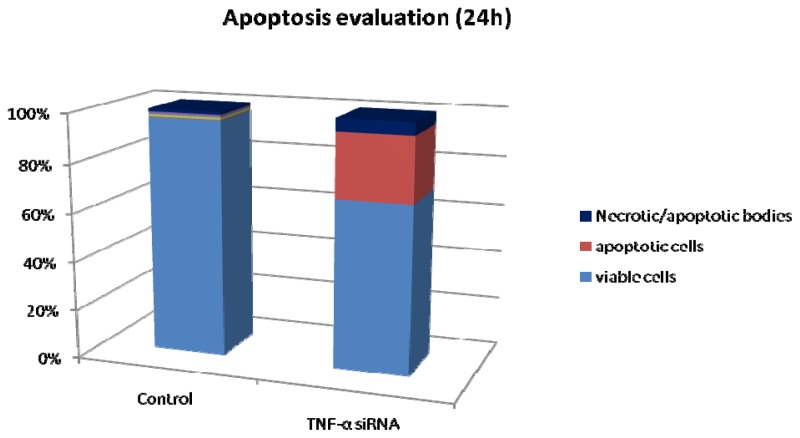
After 24 h treatment of the breast cancer cells with siRNA-TNF-α, we obtained 35.2% apoptosis and 68.8% viability compared to the control group, where the viable cells are 98.5%.

The flow cytometry results obtained indicate that blocking the expression of TNF-α in the triple negative breast cancer cell line leads to apoptosis, as we can see from Figure 2. So, after 24 h of treatment with siRNA-TNF-α, we obtained a high percentage of apoptotic cells.

### 2.3. Apoptotic Gene Expression Profile in the Hs578T Cell Line after TNF-α Gene Inhibition

PCR array technology was used to examine the transcript levels of 84 genes involved in apoptosis. After data analysis with the ΔΔ*C*_t_ method, we obtained a relevant *p*-value for 15 genes after comparing the treated with untreated groups. From these, three were down-regulated and 12 up-regulated, as we can see in Table 1. Most of the genes from Table 1 are members of the extrinsic apoptosis pathway, mediated by receptors.

RTCA is a powerful biotechnology tool suited for large scale screening [18], used in evaluating the antiproliferative effect and inhibition of migratory processed by knockout of *TNF-α*. The role of TNF-α is controversial; some investigations have proved apoptotic or necrotic effects of TNF-α, while others furnished evidence that endogenous TNF-α activates cellular growth and tumor progression [19].

In a similar study, TNF-α induced inhibition of proliferation and enhanced the expression of p21^cip/waf1^ and p27^kip1^ in human glioma cells. p21 might be regulated by NF-κB or p53 independently, as confirmed by the present experiment. The inhibition of cell proliferation does not have a direct role in rendering the cells resistant to TNF-α mediated cytotoxicity [20]. Dong *et al.* reveals that TNF-α can promote epithelial-mesenchymal transition (EMT) of MCF-7 cells and activates cell migration, being in agreement with our findings according to which down-regulation of TNF-α inhibits cell migration.

Our study supports the hypothesis that the TNF-α cytokine sustains the growth and spread of breast cancer. The constitutive synthesis of TNF-α [20] observed in many tumor types leads to the activation of cell survival signaling pathways and the synthesis of cytokines (including TNF-α), chemokines and angiogenic factors [17]. There is evidence that indicates that TNF-α is involved in the transformation, proliferation, angiogenesis, invasion and metastasis of many cancers [21]. Tumor necrosis factor-α has an important role in the tumor microenvironment, where its secretion is increased in both stromal and tumor cells [22]. This is an example of a feed-back mechanism, where the secretion of TNF-α stimulates its own formation, promoting tumor cell growth, survival, invasion, metastasis and neoangiogenesis [23,24].

After blocking the expression of TNF-α in the triple negative breast cancer cell line, our cell viability and flow citometry data suggest that cells undergo apoptosis. Down regulation of TNF-α blocks the expression of NF-κB pathway that plays a key role in tumor cell survival. Constitutive activation of the NF-κB in tumor cells [17,25] is triggered by TNF-α. Soluble TNF-α binds to TNFR1 that is highly expressed on tumor cells and determines its trimerization that internalizes the message with the recruitment of an adaptor protein, TNF-R1-associated death domain protein (TRADD). The signaling cascade continues with the phosphorylation of TRAF2 with the help of RIP (a death domain kinase) and leads to the activation of the IKK complex [26]. This leads to the degradation of IkB and the release of the active heterodimer NF-κB, which translocates to the nucleus. Here, the transcription factor induces the transcription of the target survival genes.

Our PCR array data show that removing the ligand leads to the removal of the adaptor protein as well. Down-regulation of TRADD completely blocks the cell survival and inflammation supporting pathways, leaving room for new interactions between proteins that participate in other signaling pathways to control the development of tumor cell growth or apoptosis.

The TNF-α receptor family is mediated through several regulatory factors that are pro/anti-apoptosis regulators. By inhibiting the expression of TNF-α, the cell survival pathway controlled by TNFR1 is blocked, but the apoptosis signals internalized by the TNFSF10 and TNFRSF1A are not blocked, as observed in our data, leading to the activation of JNK kinase. When NF-κB is suppressed, JNK is activated and the TNF receptor signaling pathway shifts the fate of the cell from survival toward apoptosis [27].

The *BAK-1* gene expression is also down-regulated, leading us to believe that cell apoptosis is not regulated through the cell death pathway that is mediated by mitochondria. BAK-1 increases apoptosis by accelerating the opening of the mitochondrial voltage dependent anion channel [28] and counteracts the protection from apoptosis provided by Bcl-2 [29].

There are studies indicating that TNF-α is implicated in drug resistance [30] through the activation of the NF-κB cell survival pathway leading to the inhibition of apoptosis. Other studies suggest that blocking the TNF-α expression may compromise the docetaxel chemotherapy efficiency [31]. Our results conclude that TNF-α is proving to be an efficient target in triple negative breast cancer.

Therefore, the direct targeting of TNF-α in tumor cells could be an approach to novel treatment designs. Targeting the ability of tumor cells to proliferate by blocking the intracellular cell survival pathways activated by TNF-α definitely leads tumor cells to apoptosis. At the same time, a combination with other conventional or targeted therapies might be more efficient or at least stop the tumor development and metastasis.

## 3. Experimental Section

### 3.1. Cell Culture and Treatment

In our experiment, we used Hs578T, a triple negative breast cancer cell line. Cells were cultured in Dulbecco’s Modified Eagle Medium (DMEM) with a high glucose concentration, supplemented with 10% fetal bovine serum (Sigma-Aldrich, St. Louis, MO, USA), glutamine 2 mM, penicillin 100 UI/mL and insulin. Cells were grown in a humidified 5% CO_2_ incubator at 37 °C. Cells were treated using the reverse transfection method that involves transfecting and plating the cells simultaneously. Half a million cells were seeded in a six-well plate and treated with siRNA-TNF from Silencer^®^ siRNA Transfection II Kit (Ambion, Austin, TX, USA). For each well, we used 5 μL siPORT NeoFX transfection agent dissolved in 95 mL Opti-MEM I (Gibco-Invitrogen, Paisley, UK). After 10 min of incubation at RT for each well, 2.5 μL siRNA-TNF were diluted in 97.5 μL Opti-MEM I and mixed with the transfection agent in order to achieve 50 nM in the cell culture medium. We incubated the mixture for 10 min at RT and then distributed it on the plate. Cells were cultured in a total volume of 4 mL Opti-MEM I/well for 24 h at 37 °C, 5% CO_2_ before analysis.

### 3.2. Dynamic Monitoring of Hs578T Cell Proliferation and Cell Migration Using the xCELLigence RTCA System

The breast cancer cell line Hs578T was seeded in 5000 cells/well, the optimal cell density for cell proliferation assay. The cell growth curves were recorded on the xCELLigence System in real time, every 30 min, using an E-plate 16. Cells adhere to the bottom of each well, covering the surface of the sensor that monitors cells by measuring their cell index (CI). Cell migration was dynamically recorded in real time without labeling cells. The RTCA DP instrument uses the CIM-Plate 16 for cell migration assay. A cell density of 20,000 cells/well was used in order to assess the capacity of migration from the upper chamber through the porous membrane, where the cell-sensor is embedded, into the bottom chamber of each CIM-Plate 16 well, in response to fetal serum as a chemoattractant. The impedance is correlated with a numerical increase of the cells that migrated on the underside of the membrane by measuring cell index. The CI value represents the ratio between the Rn-Rb/Rb, measured at each time point. Rn represents the cell-electrode impedance of the well with the cells, and Rb is the background impedance of the well with media alone.

### 3.3. Apoptosis by Flow Cytometry

In this study, we used a marked-antibody staining protocol from Annexin V: FITC assay kit (AbD seroTec, Oxford, UK). For each sample, we used triplicates and evaluated apoptosis after 24 h of treatment with siRNA-TNF-α. After removing the cells from the culture plates, they were resuspended in 300 mL Binding Buffer and 1 μL Annexine V-FITC and incubated for 15 min at room temperature, protected from light. Just before flow cytometry analysis, we added 1 μL of PI. The rate of apoptosis was evaluated using FACSCantoII flow cytometer, and the data obtained was analyzed with BD FACSDiva software.

### 3.4. Gene Evaluation with RT^2^Profiler™ PCRarray Technology

After 24 h from treatment, total ARN was extracted using TriReagent (Sigma-Aldrich, St. Louis, MO, USA), according to the manufacturer’s protocol. The extracted ARN was purified using the RNeasy Mini Kit (Qiagen, Hilden, Germany). ARN purity and concentration was measured using Agilent 2100 Bioanalyzer, and the spectrophotometer Nano Drop 1100. 350 μg of total ARN was reversely transcribed using the C03 Kit from Quiagen. For the PCR array analysis, we used 7 μg of cDNA for each well, and all experiments were performed in triplicates. In the array analysis, we evaluated 84 genes (the design of the PCR-array plate and gene description is presented in supplementary Tables S1 and S2) involved in apoptosis with the Human Apoptosis RT^2^Profiler™ PCR Array plate, and the PCR-array reaction was done on the LightCycler 480 instrument (Roche, Rotkreuz, Switzerland) and the PCR cycles performed according to the manufacturer instructions.

### 3.5. Network Analysis

The Ingenuity System Pathway Analysis was used to interpret the data and generate a canonical network with the available interactions among the statistically significant genes implicated in apoptosis from the 84 genes evaluated with Human Apoptosis RT^2^Profiler™ PCR Array plate.

## 4. Conclusions

The results described here indicate that the direct target of TNF-α in the Hs578T breast cancer cell line increases the levels of certain pro-apoptotic factors that modulate different cellular networks that direct the cells towards death. Therefore, this strategy that can suppress pathways involved in cell proliferation and migration has enormous tumor cell stabilization potential and also the potential to tilt the balance toward cell apoptosis.

## Supplementary Information



## Figures and Tables

**Figure 3:**
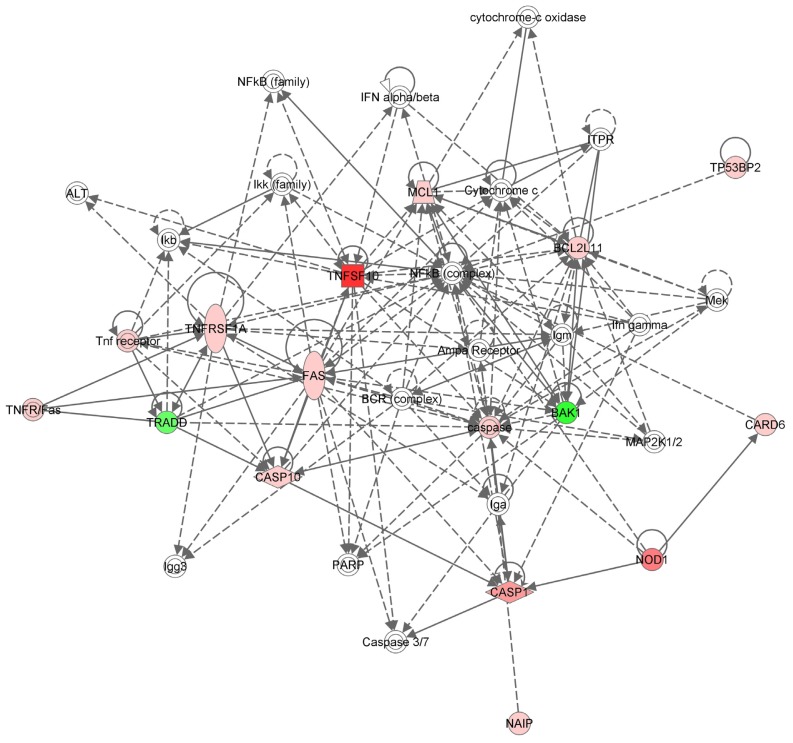
The apoptosis canonical network associated with the 15 genes involved in triple negative breast cancer *TNF-α* gene silencing generated by Ingenuity that marks the relationship between the known genes involved in apoptosis.

**Table 1:** Genes that were found to be relevant after comparing the treated with untreated groups. With green are marked the down-regulated genes and with red the up-regulated ones.

Gene	Gene symbol	Fold regulation	*p*-Value
tumor necrosis factor (ligand) superfamily, member 10	TNFSF10	4.6203	0.000025
nucleotide-binding oligomerization domain containing 1	NOD1	3.2221	0.01072
caspase 1, apoptosis-related cysteine peptidase	CASP1	1.8851	0.007098
Fas (TNF receptor superfamily, member 6)	FAS	1.7187	0.0127
tumor protein p53 binding protein, 2	TP53BP2	1.6911	0.013546
NLR family, apoptosis inhibitory protein	NAIP	1.6756	0.01393
myeloid cell leukemia sequence 1 (BCL2-related)	MCL1	1.6073	0.004842
BCL2-like 11 (apoptosis facilitator)	BCL2L11	1.5526	0.011312
tumor necrosis factor receptor superfamily, member 1A	TNFRSF1A	1.4893	0.008169
caspase recruitment domain family, member 6	CARD6	1.38	0.003481
caspase 10, apoptosis-related cysteine peptidase	CASP10	1.3485	0.028438
v-raf murine sarcoma viral oncogene homolog B1	BRAF	1.2056	0.035962
BCL2-antagonist/killer 1	BAK1	−2.1967	0.012989
tumor necrosis factor	TNF	−1.7719	0.015681
TNFRSF1A-associated via death domain	TRADD	−1.346	0.043775
