# Supplementary material for: TNF-α Gene Knockout in Triple Negative Breast Cancer Cell Line Induces Apoptosis
Source: Int J Mol Sci. 2012 Dec 24;14(1):411–20. doi: 10.3390/ijms14010411 (PMC3565271; doi:10.3390/ijms14010411)
Supplement: Supplementary file 1 [file ijms-14-00411-s001.pdf]

Supplementary Information

Table S1. Array Layout.

ABL1<br>A01	AKT1<br>A02	APAF1<br>A03	BAD<br>A04	BAG1<br>A05	BAG3<br>A06	BAG4<br>A07	BAK1<br>A08	BAX<br>A09	BCL10<br>A10	BCL2<br>A11	BCL2A1<br>A12
BCL2L1<br>B01	BCL2L10<br>B02	BCL2L11<br>B03	BCL2L2<br>B04	BCLAF1<br>B05	BFAR<br>B06	BID<br>B07	BIK<br>B08	NAIP<br>B09	BIRC2<br>B10	BIRC3<br>B11	XIAP<br>B12
BIRC6<br>C01	BIRC8<br>C02	BNIP1<br>C03	BNIP2<br>C04	BNIP3<br>C05	BNIP3L<br>C06	BRAF<br>C07	NOD1<br>C08	CARD6<br>C09	CARD8<br>C10	CASP1<br>C11	CASP10<br>C12
CASP14<br>D01	CASP2<br>D02	CASP3<br>D03	CASP4<br>D04	CASP5<br>D05	CASP6<br>D06	CASP7<br>D07	CASP8<br>D08	CASP9<br>D09	CD40<br>D10	CD40LG<br>D11	CFLAR<br>D12
CIDEA<br>E01	CIDEB<br>E02	CRADD<br>E03	DAPK1<br>E04	DFFA<br>E05	FADD<br>E06	FAS<br>E07	FASLG<br>E08	GADD45A<br>E09	HRK<br>E10	IGF1R<br>E11	LTA<br>E12
LTBR<br>F01	MCL1<br>F02	NOL3<br>F03	PYCARD<br>F04	RIPK2<br>F05	TNF<br>F06	TNFRSF10A<br>F07	TNFRSF10B<br>F08	TNFRSF11B<br>F09	TNFRSF1A<br>F10	TNFRSF21<br>F11	TNFRSF25<br>F12
CD27<br>G01	TNFRSF9<br>G02	TNFSF10<br>G03	CD70<br>G04	TNFSF8<br>G05	TP53<br>G06	TP53BP2<br>G07	TP73<br>G08	TRADD<br>G09	TRAF2<br>G10	TRAF3<br>G11	TRAF4<br>G12
B2M<br>H01	HPRT1<br>H02	RPL13A<br>H03	GAPDH<br>H04	ACTB<br>H05	HGDC<br>H06	RTC<br>H07	RTC<br>H08	RTC<br>H09	PPC<br>H10	PPC<br>H11	PPC<br>H12

Table S2. Gene Table.

Position	Unigene	GeneBank	Symbol	Description	Gene Name
A01	Hs.431048	NM_005157	ABL1	C-abl oncogene 1, non-receptor tyrosine kinase	ABL, JTK7, bcr, abl, c-ABL, p150, v-abl
A02	Hs.525622	NM_005163	AKT1	V-akt murine thymoma viral oncogene homolog 1	AKT, MGC99656, PKB, PKB-ALPHA, PRKBA, RAC, RAC-ALPHA
A03	Hs.728891	NM_001160	APAF1	Apoptotic peptidase activating factor 1	APAF-1, CED4, DKFZp781B1145
A04	Hs.370254	NM_004322	BAD	BCL2-associated agonist of cell death	BBC2, BCL2L8
A05	Hs.377484	NM_004323	BAG1	BCL2-associated athanogene	HAP, RAP46
A06	Hs.523309	NM_004281	BAG3	BCL2-associated athanogene 3	BAG-3, BIS, CAIR-1, MGC104307
A07	Hs.194726	NM_004874	BAG4	BCL2-associated athanogene 4	BAG-4, DKFZp586O2022, SODD
A08	Hs.485139	NM_001188	BAK1	BCL2-antagonist/killer 1	BAK, BAK-LIKE, BCL2L7, CDN1, MGC117255, MGC3887
A09	Hs.624291	NM_004324	BAX	BCL2-associated X protein	BCL2L4
A10	Hs.193516	NM_003921	BCL10	B-cell CLL/lymphoma 10	CARMEN, CIPER, CLAP, c-E10, mE10
A11	Hs.150749	NM_000633	BCL2	B-cell CLL/lymphoma 2	Bcl-2
A12	Hs.227817	NM_004049	BCL2A1	BCL2-related protein A1	ACC-1, ACC-2, BCL2L5, BFL1, GRS, HBPA1
B01	Hs.516966	NM_138578	BCL2L1	BCL2-like 1	BCL-XL, S, BCL2L, BCLX, BCLXL, BCLXS, Bcl-X, DKFZp781P2092, bcl-xL, bcl-xS
B02	Hs.283672	NM_020396	BCL2L10	BCL2-like 10 (apoptosis facilitator)	BCL-B, Boo, Diva, MGC129810, MGC129811
B03	Hs.469658	NM_006538	BCL2L11	BCL2-like 11 (apoptosis facilitator)	BAM, BIM, BIM-alpha6, BIM-beta6, BIM-beta7, BOD, BimEL, BimL
B04	Hs.410026	NM_004050	BCL2L2	BCL2-like 2	BCL-W, BCL2-L-2, BCLW, KIAA0271
B05	Hs.486542	NM_014739	BCLAF1	BCL2-associated transcription factor 1	BTF, KIAA0164, bK211L9.1
B06	Hs.435556	NM_016561	BFAR	Bifunctional apoptosis regulator	BAR, RNF47
B07	Hs.591054	NM_001196	BID	BH3 interacting domain death agonist	FP497, MGC15319, MGC42355
B08	Hs.475055	NM_001197	BIK	BCL2-interacting killer (apoptosis-inducing)	BIP1, BP4, NBK

Table S2. *Cont.*

Position	Unigene	GeneBank	Symbol	Description	Gene Name
B09	Hs.710305	NM_004536	NAIP	NLR family, apoptosis inhibitory protein	BIRC1, FLJ18088, FLJ42520, FLJ58811, NLRB1, psiNAIP
B10	Hs.696238	NM_001166	BIRC2	Baculoviral IAP repeat containing 2	API1, HIAP2, Hiap-2, MIHB, RNF48, c-IAP1, cIAP1
B11	Hs.127799	NM_001165	BIRC3	Baculoviral IAP repeat containing 3	AIP1, API2, CIAP2, HAIP1, HIAP1, MALT2, MIHC, RNF49, c-IAP2
B12	Hs.356076	NM_001167	XIAP	X-linked inhibitor of apoptosis	API3, BIRC4, FLJ26913, IAP-3, ILP1, MIHA, XLP2, hIAP-3, hIAP3
C01	Hs.150107	NM_016252	BIRC6	Baculoviral IAP repeat containing 6	APOLLON, BRUCE, FLJ13726, FLJ13786, KIAA1289
C02	Hs.348263	NM_033341	BIRC8	Baculoviral IAP repeat containing 8	ILP-2, ILP2, hILP2
C03	Hs.145726	NM_001205	BNIP1	BCL2/adenovirus E1B 19kDa interacting protein 1	NIP1, SEC20, TRG-8
C04	Hs.646490	NM_004330	BNIP2	BCL2/adenovirus E1B 19kDa interacting protein 2	BNIP-2, NIP2
C05	Hs.144873	NM_004052	BNIP3	BCL2/adenovirus E1B 19kDa interacting protein 3	NIP3
C06	Hs.131226	NM_004331	BNIP3L	BCL2/adenovirus E1B 19kDa interacting protein 3-like	BNIP3a, NIX
C07	Hs.550061	NM_004333	BRAF	V-raf murine sarcoma viral oncogene homolog B1	B-RAF1, BRAF1, FLJ95109, MGC126806, MGC138284, NS7, RAFB1
C08	Hs.405153	NM_006092	NOD1	Nucleotide-binding oligomerization domain containing 1	CARD4, CLR7.1, NLRC1
C09	Hs.200242	NM_032587	CARD6	Caspase recruitment domain family, member 6	CINCIN1
C10	Hs.446146	NM_014959	CARD8	Caspase recruitment domain family, member 8	CARDINAL, DACAR, DAKAR, DKFZp779L0366, FLJ18119, FLJ18121, KIAA0955, MGC57162, NDPP, NDPP1, TUCAN
C11	Hs.2490	NM_033292	CASP1	Caspase 1, apoptosis-related cysteine peptidase (interleukin 1, beta, convertase)	ICE, IL1BC, P45

Table S2. *Cont.*

Position	Unigene	GeneBank	Symbol	Description	Gene Name
C12	Hs.5353	NM_001230	CASP10	Caspase 10, apoptosis-related cysteine peptidase	ALPS2, FLICE2, MCH4
D01	Hs.466057	NM_012114	CASP14	Caspase 14, apoptosis-related cysteine peptidase	MGC119078, MGC119079
D02	Hs.368982	NM_032982	CASP2	Caspase 2, apoptosis-related cysteine peptidase	CASP-2, ICH1, NEDD-2, NEDD2
D03	Hs.141125	NM_004346	CASP3	Caspase 3, apoptosis-related cysteine peptidase	CPP32, CPP32B, SCA-1
D04	Hs.138378	NM_001225	CASP4	Caspase 4, apoptosis-related cysteine peptidase	ICE(rel)II, ICEREL-II, ICH-2, Mih1, TX, TX
D05	Hs.213327	NM_004347	CASP5	Caspase 5, apoptosis-related cysteine peptidase	ICE(rel)III, ICEREL-III, ICH-3, MGC141966
D06	Hs.654616	NM_032992	CASP6	Caspase 6, apoptosis-related cysteine peptidase	MCH2
D07	Hs.9216	NM_001227	CASP7	Caspase 7, apoptosis-related cysteine peptidase	CMH-1, ICE-LAP3, MCH3
D08	Hs.599762	NM_001228	CASP8	Caspase 8, apoptosis-related cysteine peptidase	ALPS2B, CAP4, Casp-8, FLICE, FLJ17672, MACH, MCH5, MGC78473
D09	Hs.329502	NM_001229	CASP9	Caspase 9, apoptosis-related cysteine peptidase	APAF-3, APAF3, CASPASE-9c, ICE-LAP6, MCH6
D10	Hs.472860	NM_001250	CD40	CD40 molecule, TNF receptor superfamily member 5	Bp50, CDW40, MGC9013, TNFRSF5, p50
D11	Hs.592244	NM_000074	CD40LG	CD40 ligand	CD154, CD40L, HIGM1, IGM, IMD3, T-BAM, TNFSF5, TRAP, gp39, hCD40L
D12	Hs.390736	NM_003879	CFLAR	CASP8 and FADD-like apoptosis regulator	CASH, CASP8AP1, CLARP, Casper, FLAME, FLAME-1, FLAME1, FLIP, I-FLICE, MRIT, c-FLIP, c-FLIPL, c-FLIPR, c-FLIPS
E01	Hs.249129	NM_001279	CIDEA	Cell death-inducing DFFA-like effector a	CIDE-A
E02	Hs.642693	NM_014430	CIDEB	Cell death-inducing DFFA-like effector b	-
E03	Hs.38533	NM_003805	CRADD	CASP2 and RIPK1 domain containing adaptor with death domain	MGC9163, RAIDD
E04	Hs.380277	NM_004938	DAPK1	Death-associated protein kinase 1	DAPK, DKFZp781I035

Table S2. *Cont.*

Position	Unigene	GeneBank	Symbol	Description	Gene Name
E05	Hs.484782	NM_004401	DFFA	DNA fragmentation factor, 45kDa, alpha polypeptide	DFF-45, DFF1, ICAD
E06	Hs.86131	NM_003824	FADD	Fas (TNFRSF6)-associated via death domain	MGC8528, MORT1
E07	Hs.244139	NM_000043	FAS	Fas (TNF receptor superfamily, member 6)	ALPS1A, APO-1, APT1, CD95, FAS1, FASTM, TNFRSF6
E08	Hs.2007	NM_000639	FASLG	Fas ligand (TNF superfamily, member 6)	APT1LG1, CD178, CD95-L, CD95L, FASL, TNFSF6
E09	Hs.80409	NM_001924	GADD45A	Growth arrest and DNA-damage-inducible, alpha	DDIT1, GADD45
E10	Hs.87247	NM_003806	HRK	Harakiri, BCL2 interacting protein (contains only BH3 domain)	DP5, HARAKIRI
E11	Hs.643120	NM_000875	IGF1R	Insulin-like growth factor 1 receptor	CD221, IGFIR, IGFR, JTK13, MGC142170, MGC142172, MGC18216
E12	Hs.36	NM_000595	LTA	Lymphotoxin alpha (TNF superfamily, member 1)	LT, TNFB, TNFSF1
F01	Hs.1116	NM_002342	LTBR	Lymphotoxin beta receptor (TNFR superfamily, member 3)	CD18, D12S370, LT-BETA-R, TNF-R-III, TNFCR, TNFR-RP, TNFR2-RP, TNFRSF3
F02	Hs.632486	NM_021960	MCL1	Myeloid cell leukemia sequence 1 (BCL2-related)	BCL2L3, EAT, MCL1-ES, MCL1L, MCL1S, MGC104264, MGC1839, Mcl-1, TM, bcl2-L-3, mcl1, EAT
F03	Hs.513667	NM_003946	NOL3	Nucleolar protein 3 (apoptosis repressor with CARD domain)	ARC, FLJ35304, MYP, NOP, NOP30
F04	Hs.499094	NM_013258	PYCARD	PYD and CARD domain containing	ASC, CARD5, MGC10332, TMS, TMS-1, TMS1
F05	Hs.103755	NM_003821	RIPK2	Receptor-interacting serine-threonine kinase 2	CARD3, CARDIAK, CCK, GIG30, RICK, RIP2

Table S2. ***Cont.***

Position	Unigene	GeneBank	Symbol	Description	Gene Name
F06	Hs.241570	NM_000594	TNF	Tumor necrosis factor	DIF, TNF-alpha, TNFA, TNFSF2
F07	Hs.591834	NM_003844	TNFRSF10A	Tumor necrosis factor receptor superfamily, member 10a	APO2, CD261, DR4, MGC9365, TRAILR-1, TRAILR1
F08	Hs.521456	NM_003842	TNFRSF10B	Tumor necrosis factor receptor superfamily, member 10b	CD262, DR5, KILLER, KILLER, DR5, TRAIL-R2, TRAILR2, TRICK2, TRICK2A, TRICK2B, TRICKB, ZTNFR9
F09	Hs.81791	NM_002546	TNFRSF11B	Tumor necrosis factor receptor superfamily, member 11b	MGC29565, OCIF, OPG, TR1
F10	Hs.279594	NM_001065	TNFRSF1A	Tumor necrosis factor receptor superfamily, member 1A	CD120a, FPF, MGC19588, TBP1, TNF-R, TNF-R-I, TNF-R55, TNFAR, TNFR1, TNFR55, TNFR60, p55, p55-R, p60
F11	Hs.443577	NM_014452	TNFRSF21	Tumor necrosis factor receptor superfamily, member 21	BM-018, DR6, MGC31965
F12	Hs.462529	NM_003790	TNFRSF25	Tumor necrosis factor receptor superfamily, member 25	APO-3, DDR3, DR3, LARD, TNFRSF12, TR3, TRAMP, WSL-1, WSL-LR
G01	Hs.355307	NM_001242	CD27	CD27 molecule	MGC20393, S152, T14, TNFRSF7, Tp55
G02	Hs.654459	NM_001561	TNFRSF9	Tumor necrosis factor receptor superfamily, member 9	4-1BB, CD137, CDw137, FLJ43501, ILA, MGC2172
G03	Hs.478275	NM_003810	TNFSF10	Tumor necrosis factor (ligand) superfamily, member 10	APO2L, Apo-2L, CD253, TL2, TRAIL
G04	Hs.501497	NM_001252	CD70	CD70 molecule	CD27L, CD27LG, TNFSF7
G05	Hs.654445	NM_001244	TNFSF8	Tumor necrosis factor (ligand) superfamily, member 8	CD153, CD30L, CD30LG, MGC138144
G06	Hs.654481	NM_000546	TP53	Tumor protein p53	FLJ92943, LFS1, P53, TRP53
G07	Hs.523968	NM_005426	TP53BP2	Tumor protein p53 binding protein, 2	53BP2, ASPP2, BBP, P53BP2, PPP1R13A
G08	Hs.697294	NM_005427	TP73	Tumor protein p73	P73

Table S2. *Cont.*

Position	Unigene	GeneBank	Symbol	Description	Gene Name
G09	Hs.460996	NM_003789	TRADD	TNFRSF1A-associated via death domain	Hs.89862, MGC11078
G10	Hs.522506	NM_021138	TRAF2	TNF receptor-associated factor 2	MGC:45012, TRAP, TRAP3
G11	Hs.510528	NM_003300	TRAF3	TNF receptor-associated factor 3	CAP-1, CD40bp, CRAF1, LAP1
G12	Hs.8375	NM_004295	TRAF4	TNF receptor-associated factor 4	CART1, MLN62, RNF83
H01	Hs.534255	NM_004048	B2M	Beta-2-microglobulin	-
H02	Hs.412707	NM_000194	HPRT1	Hypoxanthine phosphoribosyltransferase 1	HGPRT, HPRT
H03	Hs.728776	NM_012423	RPL13A	Ribosomal protein L13a	L13A, TSTA1
H04	Hs.592355	NM_002046	GAPDH	Glyceraldehyde-3-phosphate dehydrogenase	G3PD, GAPD, MGC88685
H05	Hs.520640	NM_001101	ACTB	Actin, beta	PS1TP5BP1
H06	N/A	SA_00105	HGDC	Human Genomic DNA Contamination	HIGX1A
H07	N/A	SA_00104	RTC	Reverse Transcription Control	RTC
H08	N/A	SA_00104	RTC	Reverse Transcription Control	RTC
H09	N/A	SA_00104	RTC	Reverse Transcription Control	RTC
H10	N/A	SA_00103	PPC	Positive PCR Control	PPC
H11	N/A	SA_00103	PPC	Positive PCR Control	PPC
H12	N/A	SA_00103	PPC	Positive PCR Control	PPC
